# Great Library Events: From Planning to Promoting to Evaluation

**DOI:** 10.5195/jmla.2022.1405

**Published:** 2022-04-01

**Authors:** Anne Marie Romano

**Affiliations:** 1 aromano@silverhillhospital.org, The Charles P. Neumann, M.D. Medical & Patient Library, Silver Hill Hospital, New Canaan, CT

Mary Grace Flaherty is an associate professor in the School of Information and Library Science at the University of North Carolina at Chapel Hill. The author's previous experiences working at academic, health research, and public library settings shaped her understanding of the various types of programs selected to be exhibited throughout the book. Library staff responsible for planning events will benefit from this timely resource. Libraries, especially public libraries, have a long history of offering various services to their communities by hosting events and programs.

This book was sent to press during the COVID-19 pandemic. With illustrated examples of successful and creative events, we come to understand that “the primary lesson learned during these unprecedented times is the need for flexibility and adaptability, no matter how carefully we have planned” (p. 17). Especially during these times, libraries have had to research alternative ways to respond to the needs of their patrons. Pivoting from in-person to virtual events in and of itself created challenges and opportunities. The sharing of ideas throughout the book makes this a must-have for event planners.

The book starts with identifying the first steps in planning library events based on providing services that meet a specific need to a defined audience. The use of tables, diagrams, graphics, checklists, and chapter summary paragraphs called recaps make this guide extremely user friendly. Practical examples from a variety of library settings are set apart in shaded text boxes on the pages. Resources with URLs are included, and the resources section at the end of the book contains over thirty recent titles to assist in expanding programming ideas. Tools such as templates make it easy to adapt one's ideas into an actionable plan.

The life cycle of a program event is illustrated in chapters dealing with planning, funding, marketing, evaluating, reporting results, and using data to make improvements. During the idea formation phase, some questions to ask involve seeking collaborators and ensuring accessibility and inclusivity. It is suggested that community assessments and being active in the community help foster program partnerships. The chapter on funding provides a comprehensive checklist and tables outlining examples in which there is no funding, limited funding, or unlimited resources. There are several examples of where to search for funding opportunities as well as budgeting tips.

The marketing and publicizing of events chapter provides successful examples that include different communication pathways targeted to specific audiences. A wide variety of programming examples are discussed that appeal to academic, medical, and public libraries. An example in this chapter includes the “Therapy dogs at the Duke University Medical Center” program, which contains a detailed description of this popular activity (p. 51).

Flaherty spends a great deal of time discussing the evaluation process and outcome measures. Different types of data to collect are illustrated in concise tables. Organizing one's results are vitally important, especially to stakeholders. Once an event has concluded, it is easy to think about moving on to the next program, but as the author points out, it is important to consider the lessons learned to ensure successful programs in the future.

The book concludes with a chapter on the life cycle of library programming, where all the key concepts throughout the book are put into action with an illustrated example of planning physical activity events for all age groups. Lastly, the timeliness of mentioning environmental sustainability is key in understanding both commitment to overall sharing of resources and in planning programs that shape the minds of future generations.

Overall, this book is thoroughly researched and provides a comprehensive guide to planning and executing programs on a variety of ideas to a wide array of audiences. I recommend this book to public, consumer health, academic, and medical library staff who desire to host successful library events.

